# Association between the presence of CRISPR-Cas system genes and antibiotic resistance in *Klebsiella pneumoniae* isolated from patients admitted in Ahvaz teaching hospitals

**DOI:** 10.1186/s12879-024-10018-7

**Published:** 2024-10-07

**Authors:** Effat Abbasi Montazeri, Morteza Saki, Mohammad Savari, Hossein Meghdadi, Sousan Akrami

**Affiliations:** 1 Infectious and Tropical Diseases Research Center, Health Research Institute, Ahvaz Jundishapur University of Medical Sciences, Ahvaz, Iran; 2 Department of Microbiology, Faculty of Medicine, Ahvaz Jundishapur University of Medical Sciences, Ahvaz, Iran; 3 Department of Microbiology, School of Medicine, Tehran University of Medical Sciences, Tehran, Iran

**Keywords:** CRISPR/Cas genes, *Klebsiella pneumoniae*, ESBL, Carbapenemase

## Abstract

**Background:**

This study aims to investigate the frequency of *cas1* and *cas3* and CRISPR1,2,3 genes in *Klebsiella pneumoniae* isolates, as well as their connection with antibiotic resistance.

**Materials and methods:**

106 *K. pneumoniae* isolates were identified by biochemical assays and PCR. The susceptibility to antibiotics was determined by Kirby–Bauer disk diffusion method. Screening of ESBLs was undertaken by using double disk diffusion and standard disk diffusion methods. The E-test and mCIM techniques was used to confirm the disc diffusion-based carbapenem resistance profiles. CRISPR-Cas system genes were identified using PCR.

**Results:**

ESBL production was found in 19% of isolates. Carbapenemase production was found in 46% of the isolates. Furthermore, the bacteria were classified as multidrug (76%), extensively drug-resistant (4%), or pan-drug-resistant (2%). When CRISPR/Cas systems were present, antibiotic resistance was lower; conversely, when they were absent, resistance was higher.

**Conclusions:**

If the CRISPR/Cas modules aren’t present, the bacteria can still acquire foreign DNA, including antibiotic resistance genes. *K. pneumoniae* isolates with a CRISPR-Cas system were less likely to carry antibiotic-resistance genes than those lacking this defense system.

**Supplementary Information:**

The online version contains supplementary material available at 10.1186/s12879-024-10018-7.

## Introduction

Plasmid insertion and other forms of horizontal gene transfer (HGT) have enhanced bacteria’s adaptability to new environments. Most archaea and many bacteria encode clustered regularly interspaced short palindromic repeats/CRISPR-associated proteins modules, which serve as adaptive defensive safeguards against the introduction of foreign DNA, such as viruses and plasmids [1]. Small direct repeats of 21–48 base pairs (bp) are assembled into CRISPR arrays, with spacer sequences of 26–72 bp of a comparable size between them [2]. CRISPR sequences are accompanied by 4–10 *cas* genes, which are highly conserved and encode cas proteins that identify and destroy the invader’s foreign genetic material [3].

It was unintentionally discovered in 1987 in the genome of *Escherichia coli*. It was then recognized in nearly all archaeal genomes and about half of the bacterial genomes, but it has yet to be found in any eukaryote [4]. The immunological response to CRISPR/Cas involves three stages: adaption, expression, and interference. During adaptation, the acquisition machinery (*cas1* and *cas2*) captures a protospacer from the invader genome and integrates it into the host chromosome’s CRISPR array to create a new spacer. The protospacer is selected based on a protospacer adjacent motif (PAM), which serves as a binding signal for cas proteins [5]. During the expression stage, RNA polymerase is used to transcribe the CRISPR array into a pre-crRNA. This is then converted into a mature crRNA and cleaved to yield individual small crRNAs, each representing a different spacer sequence [6]. During the interference stage, crRNAs engage with an effector protein or combination of proteins to search the cell for spacer sequence-compatible nucleic acid [7]. If recognized, the Cas protein’s nuclease function cleaves or destroys the foreign DNA. Cas proteins have a role in all stages of CRISPR/Cas-mediated immunity, from new spacer integration and transcript processing to nucleic acid cleavage. CRISPR-Cas systems are divided into two categories based on *cas* gene content, cas operon architecture, cas protein sequences, and the mechanisms that support the aforementioned steps. These classifications contain six primary kinds and 33 different subclasses [8].

*K. pneumoniae* is among the most common bacteria causing hospital-acquired infections and has a high prevalence of drug resistance [9]. The presence of large molecular weight plasmids is usually related to *K. pneumoniae*’s hypervirulent phenotype and multi-drug resistance (MDR) [10]. The *K. pneumoniae* chromosomes’ CRISPR-Cas systems have been classified as I-E and I-E* types. But little is known about the CRISPR-Cas systems on plasmids in Enterobacteriaceae [11]. Whether the CRISPR-Cas system in *K. pneumoniae* facilitates HGT or functions as an immune system is still a question. Antibiotic resistance is inversely related to the existence of the CRISPR and associated sequences in the DNA of certain prokaryotes including bacteria, specifically *K. pneumoniae*, according to an enormous amount of research published in the last decade [6]. Nevertheless, the findings in this particular domain have occasionally shown conflicting outcomes [12], hence highlighting the evident necessity for further investigation in this realm. Understanding the molecular events in the mechanisms of antimicrobial resistance is a cornerstone for overcoming this problem at the core level. Gaining a comprehensive understanding of this correlation can yield novel perspectives on potential pharmacological targets aimed at combating resistant infections caused by this bacterium.

There are few reports on CRISPR/Cas in Middle Eastern *K. pneumoniae* [1, 13]. The objective of the current study was to ascertain the prevalence of CRISPR/Cas in clinical isolates of *K. pneumoniae* obtained from patients admitted to the Ahvaz Jundishapur University of Medical Sciences’ four main teaching hospitals in Iran (Imam Khomeini, Golestan, Taleghani, and Razi), and its relationship with antibiotic resistance.

## Materials and methods

### Study design and area

Non-repetitive clinical isolates of *K. pneumoniae* were obtained from a variety of clinical specimens of patients referred to the four main teaching hospitals (Imam Khomeini, Golestan, Taleghani, and Razi) connected to the Ahvaz Jundishapur University of Medical Sciences in Ahvaz, Iran, for this two-year cross-sectional study (2021–2023).

### Bacterial isolates

A total of 106 *K. pneumoniae* isolates were identified from urine (*n* = 53, 50.0%), blood (*n* = 19, 17.92%), wound (*n* = 11, 10.37%), peritoneal fluid (*n* = 7, 6.60%), sputum (*n* = 6, 5.66%), and other specimens (*n* = 10, 9.43%). The sources of specimens are shown in Table 1. On sheep blood agar plates, all bacterial isolates were cultured at 35 °C. Biochemical tests such as lysine iron agar, triple sugar iron (TSI) agar, ornithine decarboxylase, and urea/citrate utilization were used to identify the isolates. Until they were needed, they were then stored at -20 °C in Lauria Bertani [8] broth containing 25% glycerol (v/v). The strain of *K. pneumoniae* ATCC^®^ 13883TM was employed as the control.

**Table 1:** Constituent ratios of wards where the samples originated from

Ward	Number of Samples (%)
Internal medicine	13 (12.26)
Intensive care unit	11 (20)
Women	13 (12.26)
Men	44 (41.50)
Surgery	6 (5.66)
Burn	8 (7.54)
Infectious diseases	4 (3.77)
Urology	11 (20)
Nephrology	1 (0.94)
Neurology	2 (1.88)
Emergency	2 (1.88)
Orthopedy	2 (1.88)
Pediatrics	2 (1.88)
Ear, nose, throat	2 (1.88)

### Isolates’ molecular confirmation

To recover genomic DNA from isolates, we used the boiling technique [14]. Briefly, the bacterial pellets were resuspended in 200 µl of TE buffer (Tris-HCl [10mM]: EDTA [1 mM]) and subjected to 15 min of boiling. Immediately after boiling, the microfuge tubes were placed in an ice bath for 15 min and then centrifuged for 5 min at 14,000 rpm at room temperature. The supernatant containing DNA (100 µl was transferred to another clean tube and stored at − 20 °C. The NanoDrop spectrometer PROMO was used to evaluate the quality and average DNA yield. The specific oligonucleotide primers for Isolates’ molecular confirmation were used as listed in Table 2. 12.5 µl of master mix (Ampliqon, Denmark), 0.5 µl of each forward and reverse primer (10 pM), 1 µl of the DNA template, and 10.5 µl of nuclease-free water were used in the PCR reactions, which had a final volume of 25 µl. The following protocol was used to conduct PCR reactions in a thermocycler (Biorad S1000 Thermal Cycler, USA): five minutes of initial denaturation at 95 °C, thirty seconds of denaturation at 95 °C for 35 cycles, thirty seconds of annealing, forty-five seconds of extension at 72 °C, and five minutes of final extension at 72 °C. Nuclease-free water and *K. pneumoniae* ATCC^®^ 13883TM were used as the positive and negative controls, respectively. Results from PCR were run on a 1.5% agarose gel and assessed in a UV lamp.

**Table 2:** Oligonucleotide primer sequences used in the study for 106 *K. pneumoniae* isolates

Gene	Primer sequence (5 ‘- 3’)	Product size (bp)	References
*16s rRNA*	F- AGCCGACCTGAGAGGGTGA R- TCTGGACCGTGTCTCAGTTCC	515	[37]
*cas1*	F- GCTGTTTGTCAAAGTTACCCGCGAACTC R- GTTTTGATCGCCTCATGAGTCACAGTTG	208	[23]
*cas3*	F- TGGCCGACATTTGATTCAGC R- CCATGCTTAACATTCATCAC	620	[23]
*I-E CRISPR1*	F-CAGTTCCTGCAACCTGGCCT R-CTGGCAGCAGGTGATACAGC	Variable	[24]
I-E* CRISPR2	F-GTAGCGAAACCCTGATCAAGCG R-GCGCTACGTTCTGGGGATG	Variable	[24]
I-E* CRISPR3	F-GACGCTGGTGCGATTCTTGAG R-CGCAGTATTCCTCAACCGCCT	Variable	[24]

### Antibiotic susceptibility testing

The antimicrobial resistance testing was performed using a disk diffusion technique on Mueller-Hinton agar (MHA) plates (Merck, Germany) as recommended by CLSI [15] for amikacin (30 µg), cefotaxime (30 µg), cefoxitin (30 µg), cefepime (30 µg), chloramphenicol (30 µg), ciprofloxacin (5 µg), ampicillin/sulbactam (10/10 µg), aztreonam (30 µg), cefazolin (30 µg), ceftriaxone (30 µg), ceftazidime (30 µg), gentamycin (10 µg), piperacillin/tazobacta (100/10 µg), trimethoprim‑sulfamethoxazole (1.25/23.75 µg), nitrofurantoin (300 µg), fosfomycin (200 µg), and tetracycline (30 µg) (MAST UK). The minimum inhibitory concentrations of imipenem, ertapenem, and meropenem were determined using the E-test method by CLSI. This was accomplished by creating a suspension of the *K. pneumoniae* isolates in sterile 0.85% saline. Before applying the antibiotic-impregnated E-test sheets, these colonies solutions were adjusted to 0.5 McFarland standard turbid and added to MHA. Following a 16–18 h incubation period at 35 °C ± 2 °C at room temperature, an inhibition ellipse that was centered along the strip appeared. The lowest inhibitory concentration was measured in micrograms per milliliter (µg/ml) at the intersection of the inhibition ellipse edge and the strip using a scale. E-tests were used to determine MICs for ertapenem (≤ 0.5 µg/ml), imipenem (≤ 1 µg/ml), and meropenem (≤ 1 µg/ml) based on CLSI. [When producing carbapenemases, Enterobacteriaceae frequently exhibit intermediate or resistant responses to one or more carbapenems, this is an indication for performing carbapenamase detection [16]]. The MICs for tigecycline and colistin were determined by broth microdilution following the breakpoints of the European Committee on Antimicrobial Susceptibility Testing 2023 (The European Committee on Antimicrobial Susceptibility Testing, 2023) [17].

### Detection of extended-spectrum β-lactamase enzyme

Using different antibiotic disks, all *K. pneumoniae* isolates were checked for the formation of ESBL enzyme [18]. High levels (co-resistance) to monobactams and third-generation cephalosporins, such as Ceftazidime zone < 22 mm, Aztreonam zone ≤ 27 mm, Cefotaxime zone, or Ceftriaxone zone ≤ 25 mm, were demonstrated by ESBL-positive bacteria. In double disk synergy test (DDST), a disk of amoxicillin-clavulanic acid AMC (30 µg) and disks of the third generation Cephalosporin antibiotic such as Ceftazidime CAZ disk (30 µg), Cefotaxime CTX (30 µg), Ceftriaxone CRO (30 µg), and Cefepime FEP (10 µg), were used together with Aztronam ATM (30 µg), each disk was placed at a distance of 20 mm apart from the inhibitor disk on a lawn culture of the resistant isolate on Mueller-Hinton Agar plate. When the inhibition zone surrounding any antibiotic disk was boosted on the side of the disk containing clavulanic acid, a distinctively shaped zone known as a “keyhole” was seen, indicating the presence of ESBL.

### Detection of carbapenemase enzyme

According to CLSI guidelines [15], all carbapenem-resistant isolates were screened for carbapenemase production using the modified carbapenem inactivation method (mCIM) [19]. In the mCIM test, 1 µL loopful of an isolate of *K. pneumoniae* was chosen from overnight blood agar (Oxoid, Basingstoke, Hampshire, UK) cultures and each isolate was then separately emulsified in 2 mL Tryptic Soy Broth (TSB) in test tubes. 10 µg meropenem disc was added to each test tube after vortexing the inoculums. Four hours were spent incubating the TSB-meropenem disc suspension in the test tubes at 37 °C. The meropenem discs were removed from each solution and placed on an MHA plate that had already been seeded with 0.5 McFarland *E. coli* ATCC 25,922. After that, these plates were incubated for 18 to 24 h at 37 °C. Zones of inhibition were measured after incubation, and isolates that showed pinpoint colonies inside 16–18 mm zones or zone widths between 6 and 15 mm were identified as carbapenemase positive.

### Molecular screening of CRISPR/Cas genes

The boiling method was used to extract the DNA from each bacterial isolate, as previously reported. Table 2 shows the PCR primers used to identify CRISPR/Cas genes. All CRISPR types underwent the following PCR cycle: initial denaturation for five minutes at 95 °C, subsequent denaturation for one minute at 94 °C, annealing for thirty seconds at 62 °C, and extension for one minute at 72 °C. Thirty-five times each was spent on the denaturation, annealing, and extension steps; the last extension step was carried out for ten minutes at 72 °C. To ensure sterility, a negative control was added in every study. In a 1X Tris/Borate/EDTA (TBE) buffer containing the DNA-safe dye, all PCR products were separated and identified by gel electrophoresis on a 1.5% agarose gel at 100 V for 1 h [20]. Ultimately, an ultraviolet transilluminator (Vilber lourmat, Marne-la-Vallée cedex 3, France) was used to confirm the expected PCR amplicon band by comparing the PCR product size to a 100 base-pair DNA ladder (Fermentas, Maryland, USA).

### Statistical analysis

Microsoft Excel and SPSS version 22 statistical software (IBM Corporation, Armonk, NY, USA) were used to analyze descriptive data. Pearson chi-square or One-tailed Fisher’s exact tests (when one or more of the cell counts is less than 5) were used to compare the occurrence of the CRISPR-Cas system and its subtypes and the antibiotic resistance among *K. pneumoniae* isolates. A P-value of less than 0.05 was regarded as significant. The findings are displayed as relative frequency-based descriptive statistics.

## Results

### Distribution of K. pneumoniae isolates

From December 2021 to November 2023, 106 *K. pneumoniae* isolates were collected from a variety of specimens. Patients had a mean age of 43.1 ± 16.3 (10–81 yearsc for men and 36.9 ± 12.7 (11–82 years) for females. Males comprised around 52.8% of the cases. The majority of male patients (35.1%) were aged 25 to 50.

### Antimicrobial susceptibility tests of K. pneumoniae isolates

Most *K. pneumoniae* isolates indicated a high ratio of resistance against studied antibiotics: ampicillin/sulbactam (100%), nitrofurantoin (45%), trimethoprim‑sulfamethoxazole (63%), ceftazidime (56%), cefotaxime (50%), tigecycline (10%), meropenem (50%), ceftriaxone (38%), ciprofloxacin (44%), imipenem (30%), piperacillin/tazobactam (27%), colistin (10%), cefepime (30%), tetracycline (32%), amikacin (6%), ertapenem (38%), Fosfomycin (6%), cefoxitin (50%), chloramphenicol (40%), aztreonam (80%), cefazolin (65%), gentamycin (36%). ESBL production was found in 19.8% (21/106) of the isolates, as well as carbapenemase production in 46.2% (49/106). ESBL-producing *K. pneumoniae* were sensitive to ertapenem (85%), tigecycline (85%), colistin (95%), piperacillin-tazobactam (61%), aztreonam (80%), and tetracycline (80%), with the exception of ampicillin, to which *K. pneumoniae* strains are inherently resistant. The carbapenemase-producing isolates were multidrug-resistant (MDR) and were only responsive to tigecycline (65%), colistin (95%), and tetracycline (81%).

### Comparative analysis of the CRISPR/Cas system components in K. pneumoniae isolates

The genes for *cas1*, *cas3*, I-E CRISPR1, I-E* CRISPR2, and I-E* CRISPR3 were found by PCR. Based on the presence or lack of genes, Table 3 displays the various profiles found in the 106 strains. Table 3 demonstrates that whereas some strains (profiles 2–6) contained just one system component—CRISPR or Cas—others (profiles 8–23) had both components. Various gene combinations were found, as Table 3 illustrates. 53% of the strains, or 57, tested negative for both Cas and CRISPR. These strains were classified as carbapenemase producing bacteria (*n* = 28) or ESBL-MDR strains (*n* = 19). Notably, the prevalence of the CRISPR/Cas system was 2 (2/49, 4.08%) in the ESBL-producing and MDR strains, also it was 21 (21/49, 42.8%) in carbapenemase-producing bacteria. Electrophoresis gel images are available in the supplementary file S1.

**Table 3:** The distribution of *cas1*,* cas3* and CRISPR genes in *K. pneumoniae* isolates

Profile	cas and CRISPR genes	No. (%)
1	**-**	57 (53.7)
2	*cas1*	1 (0.9)
3	*cas3*	11 (20)
4	I-E CRISPR1	7 (6.6)
5	I-E* CRISPR2	1 (0.9)
6	I-E* CRISPR3	1 (0.9)
7	*cas1* + *cas3*	2 (1.8)
8	*cas1* + I-E CRISPR1	2 (1.8)
9	*cas1* + I-E* CRISPR2	1 (0.9)
10	*cas3* + I-E CRISPR1	1 (0.9)
11	*cas3* + I-E* CRISPR2	1 (0.9)
12	I-E CRISPR1 + I-E* CRISPR2	1 (0.9)
13	*cas1* + *cas3* + I-E CRISPR1	1 (0.9)
14	*cas1* + Cas3 + I-E* CRISPR2	3 (2.8)
15	*cas3* + I-E* CRISPR2 + I-E* CRISPR3	1 (0.9)
16	*cas3* + I-E CRISPR1 + I-E* CRISPR2	1 (0.9)
17	*cas1* + I-E CRISPR1 + I-E* CRISPR3	2 (1.8)
18	*cas1* + I-E CRISPR1 + I-E* CRISPR2	1 (0.9)
19	*cas1* + I-E* CRISPR2 + I-E* CRISPR3	1 (0.9)
20	*cas1* + Cas3 + I-E* CRISPR2 + I-E* CRISPR3	3 (2.8)
21	*cas1* + I-E CRISPR1 + I-E* CRISPR2 + I-E* CRISPR3	1 (0.9)
22	*cas3* + I-E CRISPR1 + I-E* CRISPR2 + I-E* CRISPR3	1 (0.9)
23	*cas1* + *cas3* + I-E CRISPR1 + I-E* CRISPR2 + I-E* CRISPR3	2 (1.8)

### The CRISPR/Cas system’s frequency in nosocomial K. pneumoniae isolates and its relationship to antibiotic resistance

It has been demonstrated that the CRISPR/Cas system impedes plasmid stability and transformation, which is problematic because plasmids typically carry drug-resistant genes. Thus, we looked at whether in vitro drug resistance in clinical strains was associated with the presence of CRISPR/Cas.

Seventeen (85%) of the *cas1* PCR-positive isolates were found to have at least one CRISPR array (CRISPR1, CRISPR2, or CRISPR3) according to the PCR results. Eighteen (40%) of the carbapenem-resistant bacteria (triple resistance to ertapenem, imipenem, and meropenem) had the CRISPR/Cas system. Two (9.5%) ESBL-producing bacteria and twenty-eight (57.14%) non-ESBL strains had higher CRISPR/Cas system prevalences, suggesting a significant (p-value > 0.05) negative association between resistance and prevalence.

Drug-resistant *K. pneumoniae* has a low frequency of the CRISPR/Cas system, which suggests that it may limit the acquisition of drug-resistance genes. For CRISPR1/Cas, CRISPR2/Cas, and CRISPR3/Cas, this system was detected in 13 (68%), 16 (84%), and 11 (57%) of the sensitive isolates, respectively. By comparison, the CRISPR1/Cas, CRISPR2/Cas, and CRISPR3/Cas values for resistant isolates were 43%, 53%, and 36%, respectively.

### Relationship between the prevalence of the CRISPR/Cas system and drug resistance

Compared to CRISPR/Cas-positive isolates, the percentage of *K. pneumoniae* isolates resistant to antibiotics was higher in CRISPR/Cas-negative isolates. The CRISPR/Cas-positive isolates were resistant to amikacin (48%), ampicillin/sulbactam (57%), aztreonam (42%), cefoxitin (51%), chloramphenicol (26%), gentamycin (46%), tetracycline [10] and piperacillin/tazobactam (51%). In contrast, the resistance rates to those medications in CRISPR/Cas-negative isolates were 61%, 70%, 43%, 68%, 78%, 63%, 22%, and 52%, respectively, in isolates lacking the CRISPR/Cas system. CRISPR absence is more prevalent in resistant strains, however not statistically significant.

Our findings indicated that CRISPR1 was most common in sensitive strains without MDR phenotype (*n* = 11; 61.1%), followed by CRISPR3 (*n* = 5; 27.7%), and then CRISPR2 (*n* = 4; 22.2%). Resistance bacteria are more likely to have CRISPR2 (*n* = 13; 41.9%), CRISPR1 (*n* = 11; 35.4%), and CRISPR3 (*n* = 8; 25.8%). According to Table 4, the strains belonging to the MDR, extensively-drug resistant (XDR) and pan-drug resistant (PDR) groups exhibited the most differences, with most of them missing CRISPR/Cas systems. The term “multidrug-resistant” (MDR) strain is used to describe bacteria that are resistant to at least one antibiotic across three or more chemical families. The term “extensively drug-resistant” (XDR) strain describes bacterial isolates that are resistant to at least one antibiotic agent in all but one or two antimicrobial subcategories. The term “pan drug-resistant” (PDR) refers to bacteria that are resistance to all tested antibiotics in all classes available in the medical community [21].

**Table 4:** Drug resistance pattern between CRISPR/Cas-positive and -negative isolates of *K. pneumoniae*

MDR/XDR/PDR	Number of Strains (%)	CRISPR/Cas-Positive No. (%)	CRISPR/Cas-Negative No. (%)
**MDR**	81 (76)	31 (38)	50 (16)
**XDR**	5 (4)	2 (4)	3 (5)
**PDR**	3 (2)	1 (2)	2 (3)
**Non- MDR/XDR/PDR**	17 (16)	15 (30)	2 (3)

## Discussion

Recent research has linked the existence of CRISPR/Cas systems to increased antibiotic sensitivity and plasmid uptake in several bacterial species [12, 22]. Few research on this problem have been carried out in the Middle East thus far. In a study of Kadkhoda et al. [1], 34.2% of *K. pneumoniae* isolates were found to harbor the CRISPR-Cas system. The relatively low prevalence of CRISPR systems in that study can be attributed to the fact that most of the strains contained antibiotic-resistant genes; therefore, these isolates did not have CRISPR systems [1]. Jwair et al. [13] in Iraq demonstrated that there was a highly significant inverse association between the pr10evalence of CRISPR-Cas system and drug resistance in carbapenem-resistant and ESBL-producing *K. pneumoniae*.

In this study, we aimed to assess the prevalence of the CRISPR-Cas systems and their association with antibiotic resistance in one of the most challenging bacterial pathogens, *K. pneumoniae*. Two Cas genes [23] and three CRISPR genes [24] were utilized to test CRISPR/Cas systems using PCR. In this article, the distribution and type of CRISPR-Cas system in *Klebsiella* were investigated. Approximately half of the strains collected included the CRISPR-Cas system, with the majority belonging to type I-E*. Other researchers observed similar findings. The I-E* strain was dominant in the *K. pneumoniae* isolates in previous research [25, 26]. Like the finding of Hu et al., IE and I-E* co-exist in 9 tested isolates [26]. CRISPR/Cas was detected in 49/106 (46%) clinical strains of *K. pneumoniae* isolated from hospital settings. The incidence of the CRISPR/Cas system is variable, ranging from 30.7% (54/176) to 12.4% (27/217) [23, 24, 27].

ESBL-producing *K. pneumoniae* were sensitive to ertapenem (85%), tigecycline (85%), colistin (95%), piperacillin-tazobactam (61%), aztreonam (80%), and tetracycline (80%). The carbapenemase-producing isolates were sensitive to tigecycline (65%), colistin (95%), and tetracycline (81%). In 76% and 4% of the isolates, respectively, MDR and XDR phenotypes were found. Globally, *K. pneumoniae* has a large incidence of MDR and XDR phenotypes, which has been regularly observed [4, 28–31]. Tetracycline resistance was found to be low (55%) indicating that these antibiotics may be able to substitute colistin and tigecycline against MDR and XDR *K. pneumoniae* strains, as recommended [32]. For the CRISPR/Cas-positive isolates, 49/106 (46%) were MDR (63%) or XDR (10%), and susceptibility to various antimicrobial drugs varied between the CRISPR/Cas-positive and CRISPR/Cas-negative pathogens. CRISPR/Cas-negative isolates showed similar or greater resistance to all β-lactams compared to others. A similar result was observed with amikacin, ciprofloxacin, and trimethoprim/sulfamethoxazole. Meanwhile, tetracycline, tigecycline, and colistin showed reduced resistance. This was not significant for any of the drugs studied. Wang et al. [24] and Alkompoz et al. [4] revealed similar, but statistically significant, results. Alkompoz et al. discovered that the CRISPR/Cas-positive isolates exhibited complete resistance to ciprofloxacin and carbapenems [4]. Comparably, another study conducted in the US found that all *K. pneumoniae* strains that had CRISPR/Cas were sensitive to carbapenems and all other antibiotics that had been tested [33], contradicting the substantial carbapenem resistance observed in the CRISPR/Cas-bearing isolates in the current investigation. When we investigated the relationships between antimicrobial resistance and I-E and I-E* CRISPR/Cas, we discovered that isolates carrying I-E were more sensitive to tested antimicrobial agents than those carrying I-E*. Furthermore, a previous study on 176 clinical isolates from Taiwan discovered that, in comparison to I-E*, I-E was associated with higher sensitivity to gentamicin, trimethoprim/sulfamethoxazole, and cefotaxime [34].

Numerous studies revealed that *K. pneumoniae*’s CRISPR/Cas systems are classified as type I-E and I-E* [35]. Apart from the highly conserved *cas1* and *cas2*, *cas3* is a crucial part of type I CRISPR/Cas, exhibiting unique DNase and helicase properties. Eight Cas genes—*cas1*,* cas2*,* cas3*,* cas5*,* cas6*,* cas7*,* cse1*, and *cse2*—are shared by both type I-E and type I-E*. The type I-E *cas3* gene is located downstream of *cas7*-*cas5*, which in type I-E* is located upstream of *cas7-cas5* [36]. Based on PCR data, we found that *K. pneumoniae* isolates include the CRISPR/Cas system, which includes *cas1* and *cas3* as well as CRISPR arrays. *cas1* and *cas3* were co-existent in 11 of our 106 samples, including 8 CRISPR2-positive isolates and 5 CRISPR3-positive isolates. The 21 isolates were categorized as subtype I-E* CRISPR/Cas system, as was previously mentioned. Antimicrobial sensitivity in Pseudomonas aeruginosa, *K. pneumoniae*,* E. coli*, and *Streptococcus pyogenes* is associated with CRISPR-Cas systems [26]. The distribution of type I-E CRISPR and antimicrobial susceptibilities in *K. pneumoniae* did not correlate with antibiotic resistance, while a previous research discovered an inverse relationship between the type I-E* CRISPR-Cas system and antibiotic resistance [34]. According to our research, compared to CRISPR-negative isolates, isolates carrying the CRISPR-Cas system showed increased sensitivity to carbapenems.

## Conclusion

The results of this study indicate that it is crucial to continuously monitor the proportion of *K. pneumoniae* type I-E and type I-E* strains, as this may affect the organism’s global patterns of development and evolution of multidrug resistance. Our findings revealed that *K. pneumoniae* isolates that express the CRISPR/Cas system are likely to have less antibiotic resistance than CRISPR-negative isolates. Analysis of the correlation between the CRISPR-Cas system and antibiotic resistance will help to identify and better understand the mechanism of bacterial resistance and provide new instructions for the prevention and treatment of bacterial antibiotic resistance. Therefore, the CRISPR-Cas system along with other genetic markers could be used for infection control by resistant pathogens, to give insights into their genetic contents and phenotypic characteristics, and also to differentiate low-risk strains of pathogens from high-risk strains.

## Electronic supplementary material

Below is the link to the electronic supplementary material.

Supplementary Material 1

## Data Availability

All data is in article.
