# Supplementary material for: Association between the presence of CRISPR-Cas system genes and antibiotic resistance in Klebsiella pneumoniae isolated from patients admitted in Ahvaz teaching hospitals
Source: BMC Infect Dis. 2024 Oct 7;24:1117. doi: 10.1186/s12879-024-10018-7 (PMC11460096; doi:10.1186/s12879-024-10018-7)
Supplement: Supplementary file 1 — Supplementary Material 1 [file 12879_2024_10018_MOESM1_ESM.docx]

**Figure 1.** Presence of (**A**) *cas1* and (**B**) *cas3* genes by PCR. M indicates the DNA marker. The numeric characters represent the sequential number of different *K. pneumoniae* isolates.


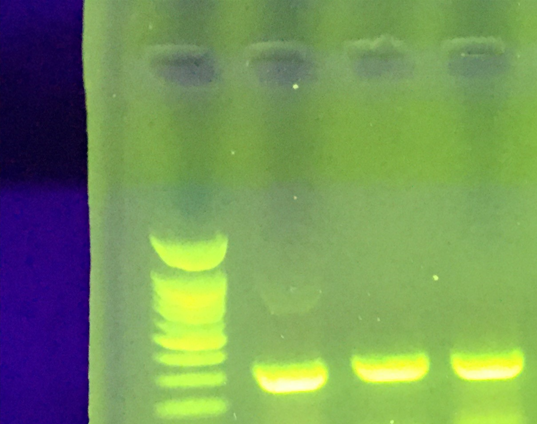


M

19

22

15

**A**

1000bp

500bp

200bp

100bp


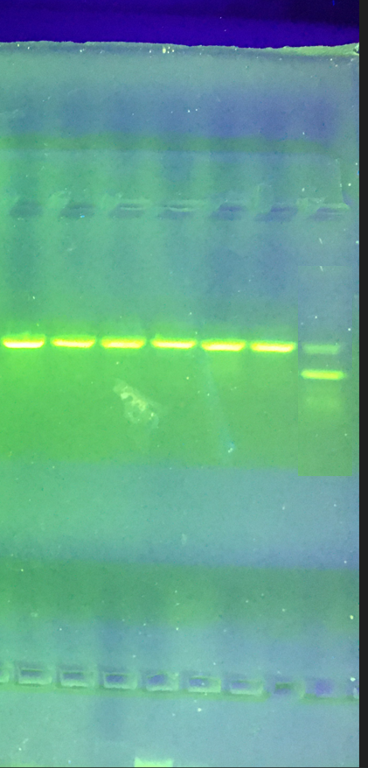


**B**

51

29

23

22

19

15

M

600bp

500bp

100bp

**Figure 2**. Presence of CRISPR genes by PCR. M indicates the DNA marker.


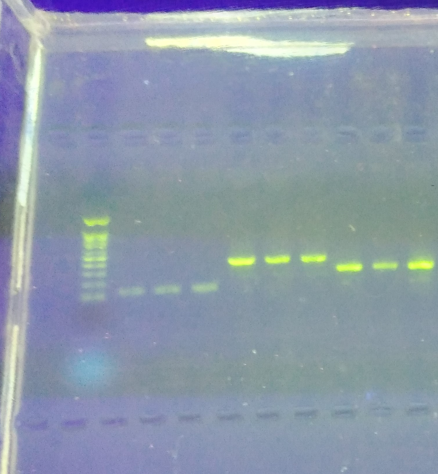


M

*I-E CRISPR1*

*I-E* CRISPR3*

*I-E* CRISPR2*
